# Real-World Application of Microscope-Integrated 400 kHz Swept-Source Intraoperative OCT in Ophthalmic Surgery

**DOI:** 10.3390/jcm15124791

**Published:** 2026-06-20

**Authors:** Xifang Zhang, Shuang Liu, Jing Guo, Shuai Yang, Tengteng Yao, Yuheng Zhang, Zhaoyang Wang

**Affiliations:** 1 Beijing Tongren Eye Center, Beijing Tongren Hospital, Capital Medical University, No. 1 Dongjiaominxiang Alley, Dongcheng District, Beijing 100730, China; joycexifang@aliyun.com (X.Z.); drguoj@163.com (J.G.); yangshuai_sh@163.com (S.Y.); yaotengteng@mail.ccmu.edu.cn (T.Y.); zhangyuheng3362@163.com (Y.Z.); 2 Beijing Luhe Hospital, Capital Medical University, Beijing 101199, China; liushuang_0313@126.com

**Keywords:** swept-source OCT, intraoperative OCT, ophthalmic surgery, intraocular tumors, choroidal melanoma

## Abstract

**Objectives**: We aimed to descriptively evaluate the feasibility and clinical utility of TowardPi BO (4K ultra-HD microscope integrated with a 400 kHz swept-source intraoperative optical coherence tomography (SS-iOCT) system) in managing various ophthalmic surgical conditions in a real-world setting. **Methods**: We analyzed surgical videos and data from 123 consecutive cases that underwent elective surgery with the assistance of this SS-iOCT system at Beijing Tongren Hospital between 2 September 2025 and 10 February 2026. Cases were included when the iOCT provided critical, real-time information that directly influenced surgical decision-making or technique modification. Cases were excluded if iOCT served only routine confirmatory or educational purposes without altering the surgical plan. **Results**: A total of 72 surgical cases were included, comprising 7 intraocular lens implantations with ciliary sulcus fixation, 19 macular holes, 3 cases of macular hole retinal detachment (MHRD), 4 cases of macular schisis with or without foveal detachment (MSRD), 12 cases of submacular hemorrhage, 20 cases of rhegmatogenous retinal detachment (RRD), and 7 intraocular mass lesions. The 400 kHz SS-iOCT significantly aided in surgical visualization, guided real-time decision-making, and prompted modifications in surgical techniques. **Conclusions**: To our knowledge, this is the first real-world study to evaluate the application of a 400 kHz SS-iOCT system across a wide spectrum of ophthalmic conditions, including its novel use in intraocular tumors. From routine to complex surgical cases, SS-iOCT enhances surgical precision and facilitates real-time decision-making, ultimately contributing to improved surgical outcomes.

## 1. Introduction

The introduction of optical coherence tomography (OCT) has profoundly transformed both the diagnosis and management of retinal diseases by providing non-invasive, high-resolution, cross-sectional images of retinal structures since 1991 [1]. The first microscope-integrated intraoperative OCT research system was demonstrated in 2010, by coupling a spectral-domain OCT (SD-OCT) device into the infinity space between the eyepiece and the objective of a commercial surgical microscope [2].

In 2013–2014, commercialized products such as the Leica Enfocus and Zeiss Rescan 700 were established, heralding the technology’s arrival as a mature feature for ophthalmic surgical microscopes. However, these systems all utilized SD-OCT, which has limited imaging speed and depth [3]. Lu et al. in 2018 demonstrated a 400 kHz SS-OCT intraoperative system employing post-objective injection of the OCT light; however, this system lacked real-time visualization [4].

Ophthalmic microsurgery is traditionally performed using stereomicroscopes and requires visualization and manipulation of sub-millimeter tissue structures with limited contrast. While there has been substantial progress in both research and commercialization efforts to bring OCT imaging into live surgery, its use is still somewhat limited due to factors such as low imaging speed, limited scan configurations, and suboptimal data visualization [5].

Recently implemented at our institution, a microscope-integrated 400 kHz swept-source intraoperative OCT (SS-iOCT) system enables real-time, high-resolution visualization of both anterior and posterior segment structures. We report its clinical utility by analyzing a series of 72 real-world surgical cases, detailing its application and discussing the specific intraoperative benefits it provides.

## 2. Materials and Methods

In this observational study, we analyzed surgical videos and data from 123 consecutive cases that underwent elective surgery with the assistance of this SS-iOCT system at Beijing Tongren Hospital between 2 September 2025 and 10 February 2026. 

Inclusion criteria: (a) complete surgical video and data available; (b) iOCT imaging was actively used by the surgeon to address a specific intraoperative question; (c) iOCT findings led to a documented intraoperative decision change or technique modification.

Exclusion criteria: cases were excluded if iOCT was used only for routine visualization, documentation, or teaching purposes without influencing the surgical course.

A total of 72 cases met the inclusion criteria, comprising 7 intraocular lens implantations with ciliary sulcus fixation, 19 macular holes, 3 cases of macular hole retinal detachment (MHRD), 4 cases of macular schisis with or without foveal detachment (MSRD), 12 cases of submacular hemorrhage, 20 cases of rhegmatogenous retinal detachment (RRD), and 7 intraocular mass lesions. Excluded cases (*n* = 51) consisted primarily of tractional retinal detachment (*n* = 10), silicone oil removal (*n* = 12), epiretinal membranes (*n* = 18) and cataract (*n* = 11).

This study followed the tenets of the Declaration of Helsinki for research involving human subjects. Informed consent was obtained from all subjects to use the data for the study.

### 2.1. Image Selection and Evaluation

All surgeries were performed by a single expert surgeon (Wang ZY) using the 4K ultra-HD microscope(TowardPi, Beijing, China) integrated with a 400 kHz SS-iOCT system. The SS-iOCT operates at a wavelength of 1060 nm and provides an A-scan depth of 12 mm, an axial resolution of 7 µm (at 12 mm depth), and a scan length of 20 mm. This SS-iOCT system offers multiple scan modes, including single-line scan, cross-line scan, five-line scan, radial scan, 3D scan, and 4D scan. To ensure both efficiency and practicality during surgery, a cross-line scan was sufficient for most cases. By adjusting its position and depth, clear and interpretable images could be obtained. Alternative modes, such as single-line dynamic scanning, were reserved for a small subset of cases requiring real-time monitoring of specific surgical events.

During surgery, intraoperative OCT provided the surgeon with clear visualization and guided surgical decision-making. For the fellow surgeons participating in the procedure, it additionally served a demonstrative and educational purpose. After the surgery, two physicians (Zhang XF and Liu S) selected the clearest surgical video frames and OCT images from the recorded footage for presentation in the Section 3.

### 2.2. Statistical Analysis

The collected data encompassed patient demographics, surgical indications, procedure types, and specific intraoperative maneuvers performed. Statistical analysis was conducted using the Statistical Package for the Social Sciences (SPSS, version 27.0; IBM Corp., Chicago, IL, USA). Numerical variables are presented as mean ± standard deviation (SD), while categorical variables are expressed as frequencies and percentages. No advanced statistical analysis was performed. Additionally, the surgeon provided a qualitative assessment of the intraoperative images.

## 3. Results

Among 72 patients, 39 were male (54.2%) and 33 were female (45.8%), with a mean age of 55.8 years (range: 18–86 years).

### 3.1. Application in Anterior Segment Surgery

**Figure 1:**
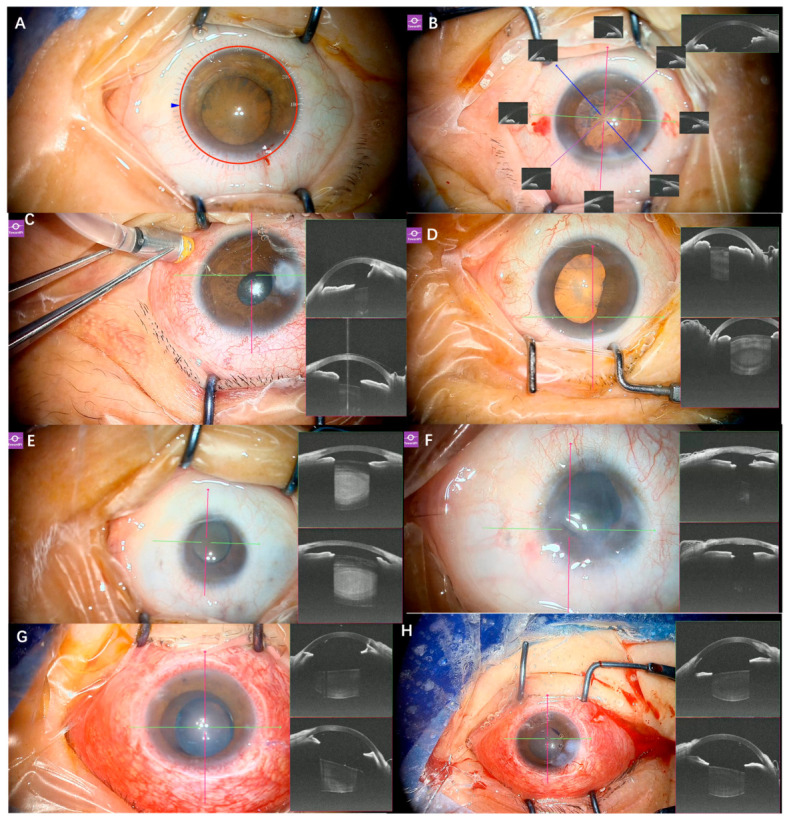
Application of real-time SS-iOCT in anterior segment surgery. (**A**) The microscope is equipped with a “Mendez Ring” for astigmatism correction and IOL ciliary sulcus fixation. (**B**) In addition to the standard line-scan mode, it can display the angle structures at eight clock hours during anterior segment surgery. (**C**) iOCT demonstrates the layered structure of the lesion in a 33-year-old male MHRD patient with adherent leucoma, facilitating teaching and illustrating the iris–cornea relationship. (**D**) iOCT reveals posterior synechiae during phacovitrectomy in a 66-year-old female with RRD. (**E**) A 47-year-old female patient with retinal detachment secondary to coloboma of the choroid, microcornea (diameter 7 × 6 mm), and nuclear cataract graded N4. Anterior segment OCT was used to observe the anterior chamber structures and assist in determining the location of the corneal incision. Based on the assessment of anterior segment OCT, the angle structure was like that of a normal cornea, and a clear corneal incision was performed. (**F**) SS-iOCT confirmed that the corneal incision was appropriately positioned in the same patient. (**G**) In a 33-year-old male with recurrent retinal detachment, SS-iOCT revealed angle adhesions and silicone oil droplets in the anterior chamber. (**H**) Goniosynechialysis was performed with a viscoelastic agent, and OCT confirmed the angle had been re-opened after its removal in the same patient. Lines of different colors correspond to the respective OCT images. The same labeling scheme applies to all subsequent images.

In anterior segment surgery, SS-iOCT visualizes the “Mendez Ring” and reveals peripheral angle structures. In cases involving anterior segment anomalies, it clearly visualizes corneal, iris, and angle abnormalities, allowing immediate confirmation of surgical outcomes after peripheral synechialysis (Figure 1).

### 3.2. Application in Cataract Surgery

**Figure 2:**
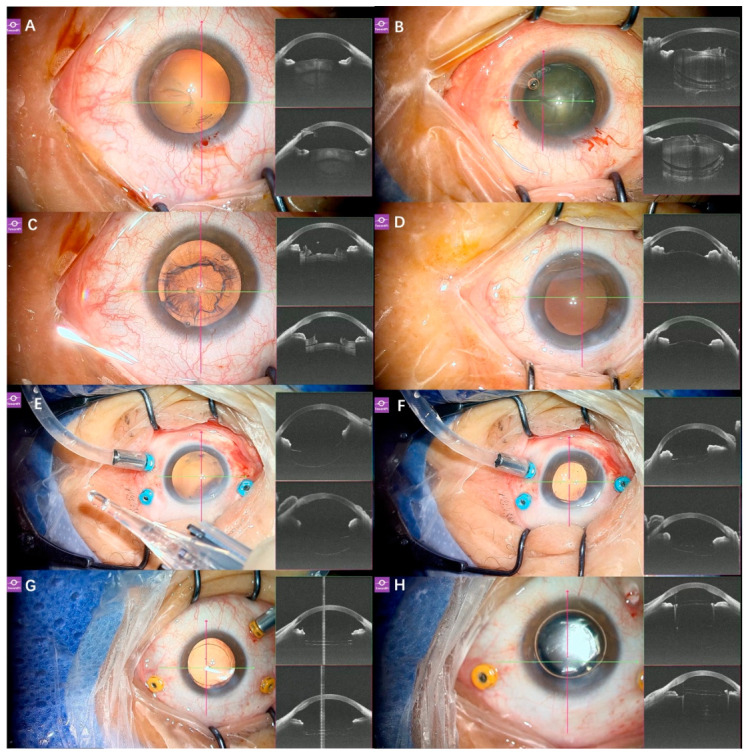
Application of real-time SS-iOCT in cataract surgery. (**A**) In a 79-year-old male patient with vitreomacular traction (VMT) undergoing phacovitrectomy, SS-iOCT clearly visualized the corneal incision. (**B**) For this 72-year-old PCV patient undergoing phacovitrectomy, SS-iOCT showed clear images following hydrodelineation (golden ring under the microscope) and hydrodissection. (**C**) In the same patient as in A, the anterior surge of the cortex, posterior capsule, and anterior vitreous following phacoemulsification indicated intraoperative disturbance of the vitreous during cataract surgery. (**D**) In a 65-year-old female patient with a macular hole (MH) undergoing phacovitrectomy, following cortical aspiration, OCT revealed anterior vitreous prolapse and apposition of the anterior and posterior capsules, despite a watertight incision and normal anterior chamber depth. This indicated that the vitreous had been disturbed during the cataract surgery. (**E**) In this 77-year-old female patient with LMH undergoing phacovitrectomy, prior to IOL insertion, OCT clearly demonstrated the capsular bag being expanded with a viscoelastic agent, facilitating IOL implantation. (**F**) In the same patient, after IOL implantation and aspiration of the viscoelastic agent from the capsular bag, posterior capsule folds were observed under both the microscope and OCT. (**G**) In the same patient as in A, after the IOL was implanted in the capsular bag, OCT confirmed the IOL was well-positioned. (**H**) Following fluid–air exchange, OCT was still able to visualize the IOL position despite the presence of gas in the anterior chamber in the same patient.

During phacovitrectomy surgery, SS-iOCT clearly delineates surgical steps, proving especially beneficial for teaching. This includes visualizing corneal incisions, hydrodissection and hydrodelineation, observing anterior and posterior capsule positions, and intraocular lens (IOL) implantation. Moreover, even when air bubbles in the anterior chamber obscure the IOL position to the naked eye following combined surgery with air–fluid exchange, SS-iOCT can accurately determine the IOL location (Figure 2).

### 3.3. Application in IOL Ciliary Sulcus Fixation

**Figure 3:**
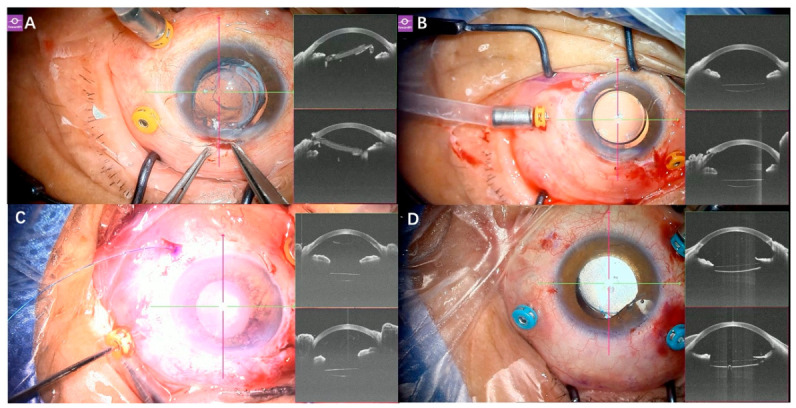
Application of real-time SS-iOCT in IOL ciliary sulcus fixation surgery. (**A**) In a 57-year-old patient with a dislocated IOL in the vitreous cavity, SS-iOCT confirmed that the IOL and capsular tension ring were removed through a clear corneal incision without contacting the corneal endothelium. (**B**) Following the Yamane technique, SS-iOCT confirmed that the IOL was well-positioned in the same patient. (**C**) In a 62-year-old female patient undergoing IOL ciliary sulcus fixation with prolene sutures, intraoperative adjustments were made based on the IOL position along two perpendicular meridians as visualized by SS-iOCT. This helped ensure optimal IOL positioning and minimize postoperative astigmatism. (**D**) In a 59-year-old male patient, SS-iOCT demonstrated the immediate postoperative result following combined Yamane technique and iris suture, confirming that the IOL was well-positioned.

SS-iOCT was used to assist intraoperative surgical guidance in seven patients (9.7%) undergoing IOL ciliary sulcus fixation. Among them, three were male and four were female. The mean age was 61.3 years (range: 42–73 years), and the mean axial length was 29.14 mm (range: 28.14–32.95 mm). SS-iOCT enabled real-time monitoring of manipulations within the anterior chamber, helping to prevent inadvertent contact between surgical instruments or a dislocated IOL and the corneal endothelium, thereby reducing the risk of corneal endothelial damage. During IOL fixation, adjustments were made based on the IOL position along two perpendicular meridians to ensure optimal IOL placement and minimize postoperative astigmatism (Figure 3).

### 3.4. Application in Rhegmatogenous Retinal Detachment (RRD)

Twenty (27.8%) eyes diagnosed with rhegmatogenous retinal detachment (RRD) were treated. Among them, 14 were male and 6 were female, with a mean age of 44.9 years (range: 18–66 years) and a mean axial length of 27.6 mm (range: 23.14–33.17 mm).

In one patient undergoing scleral buckling with a 25G endoilluminator, intraoperative OCT revealed shallow retinal detachment in the inferior degenerative area outside the primary detachment zone, guiding intraoperative adjustment of the buckling extent and contributing to improved primary success rate.

In RRD surgeries associated with pathological myopia, intraoperative OCT assisted in evaluating macular structure against a background of diffuse posterior pole atrophy, determining the presence of a macular hole and the status of the macula, thereby guiding the decision to perform either internal limiting membrane (ILM) peeling or fovea-sparing ILM peeling.

**Figure 4:**
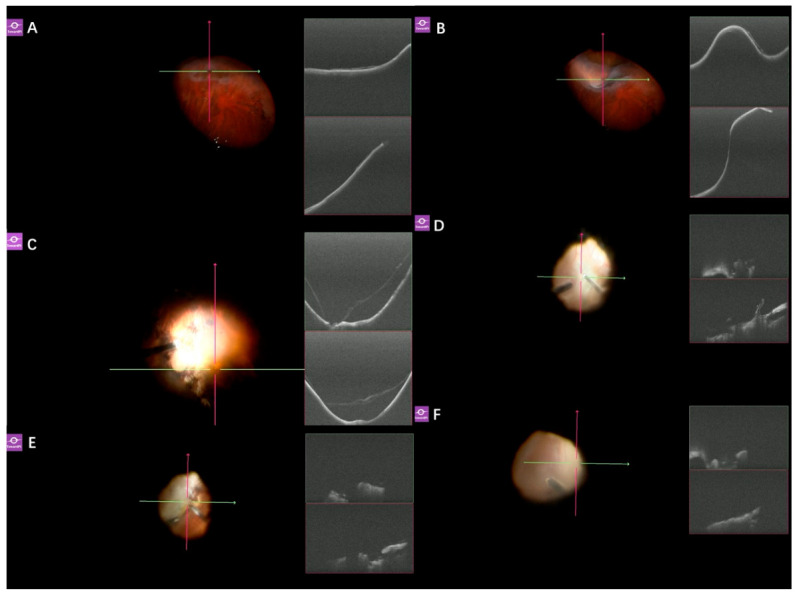
Application of real-time SS-iOCT in RRD surgery. (**A**) In an 18-year-old male patient with RRD undergoing 25G endoilluminator-assisted scleral buckling, in addition to a definite superior retinal break and detachment, there was an area of peripheral degeneration inferiorly where the presence of retinal detachment was difficult to determine. SS-iOCT revealed that the inferior degenerative area was associated with shallow retinal detachment. (**B**) Despite scleral indentation, shallow retinal detachment persisted. Therefore, an external buckle was also applied to the inferior quadrant during surgery in the same patient. (**C**) A 48-year-old female patient with RRD presented with extensive preoperative retinal detachment that precluded evaluation of macular structure from pre-operative imaging. With an axial length of 30.12 mm and diffuse posterior pole atrophy, it was difficult to determine whether a macular hole was present. Performing ILM peeling in the setting of macular thinning would increase the risk of postoperative macular hole formation. SS-iOCT confirmed the absence of a macular hole, and consequently, ILM peeling was not performed during surgery. (**D**) In a 47-year-old female patient with choroidal coloboma associated retinal detachment, SS-iOCT facilitated the identification of structures within the colobomatous area, including the optic disc and prepapillary glial tissue located in the region of the choroidal coloboma. (**E**) In the same patient, after perfluoro-n-octane injection, the optic disc appeared clearer under the microscope compared to its detached state, and OCT was able to clearly visualize the optic disc. (**F**) In the same patient, due to the absence of a typical highly reflective RPE layer within the choroidal coloboma area, the structures were difficult to identify.

In RRD associated with chorioretinal coloboma, retinal structures within the colobomatous area—including critical landmarks such as the optic disc, macula, and retinal breaks—were frequently indistinguishable. iOCT provided real-time imaging, allowing detailed scanning and observation of targeted regions beyond the capabilities of preoperative conventional OCT. However, structural identification in colobomatous eyes remains challenging and warrants further investigation (Figure 4).

### 3.5. Application in Primary or Recurrent Macular Hole (MH)

Nineteen eyes (26.4%) diagnosed with primary or recurrent macular hole (MH) were treated. Among them, 7 were male and 12 were female, with a mean age of 64.9 years (range: 48–77 years), a mean axial length of 25.3 mm (range: 22.32–34.29 mm), and a mean MH diameter of 533.5 µm (range: 128–2227 µm). Postoperatively, one MH failed to close, yielding a primary closure rate of 95%.

In primary MH, the value of intraoperative OCT (iOCT) was primarily educational. However, in refractory or recurrent MH, iOCT provided critical intraoperative guidance for determining the precise positioning of the internal limiting membrane (ILM) flap insertion, with adequate ILM insertion contributing to improved hole closure rates.

**Figure 5:**
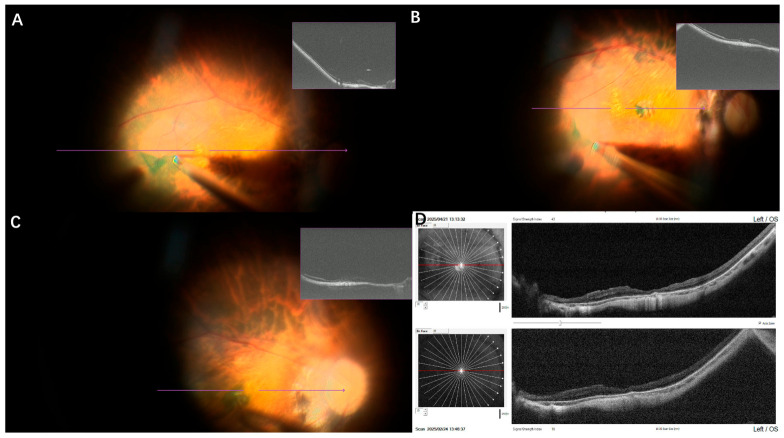
Application of real-time SS-iOCT in a 58-year-old female with a macular hole measuring 921 μm in diameter and an axial length of 30.12 mm, who underwent PPV with ILM insertion technique. (**A**) The margins of the hole appeared rigid, without significant elevation. (**B**) An ILM flap was created and plugged into the macular hole. SS-iOCT revealed the inserted ILM flap positioned on the temporal side of the hole. (**C**) Another ILM flap was created and inserted into the nasal side of the macular hole to improve the macular hole closure rate. (**D**) The macular hole closed three months postoperatively (February 2025).

In lamellar macular hole, iOCT offered surgeons additional anatomical information, facilitating assessment of macular configuration and serving as a valuable teaching tool (Figure 5).

**Figure 6:**
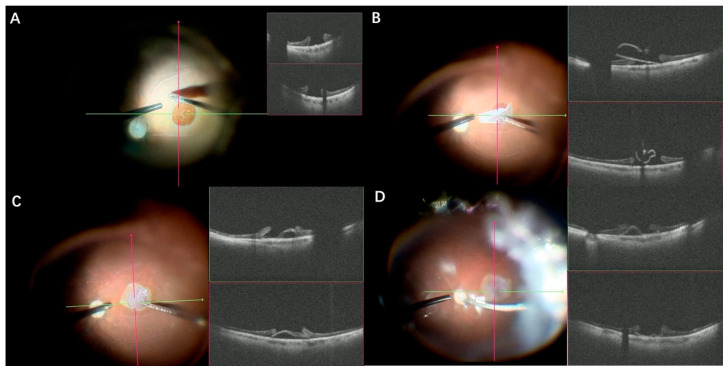
Application of real-time SS-iOCT in a 66-year-old female with a macular hole measuring 2227 μm in diameter and an axial length of 22.32 mm, who underwent PPV with amniotic membrane insertion technique. (**A**) The margins of the hole appeared rigid. (**B**) The amniotic membrane was trimmed and then plugged into the macular hole without touching the RPE. (**C**) The nasal side had already been inserted into the hole. (**D**) The inserted amniotic membrane remained visible after fluid–air exchange.

In the surgical management of a giant recurrent macular hole (2227 µm) with amniotic membrane insertion technique, iOCT clearly demonstrated the insertion process, and macular architecture remained well visualized following gas–fluid exchange (Figure 6).

### 3.6. Application in Myopic Traction Maculopathy (MTM)

**Figure 7:**
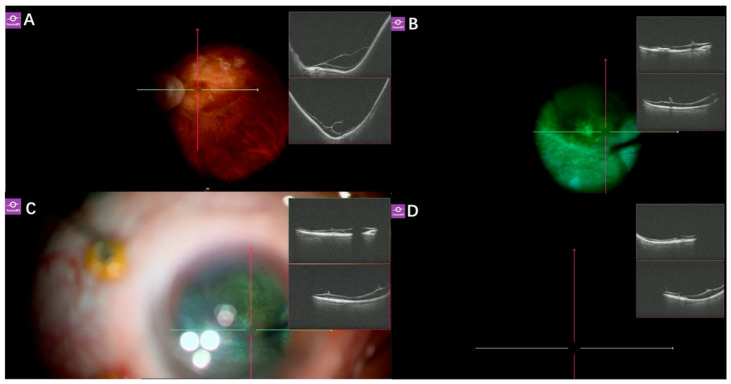
Application of real-time SS-iOCT in a 79-year-old male with VMT and an axial length of 34.29 mm, who underwent phacovitrectomy. (**A**) In an eye with an axial length of 34 mm, SS-iOCT was still able to clearly visualize macular schisis and vitreous traction. (**B**) Even with ICG staining present and not yet aspirated, OCT was still able to produce clear images. (**C**,**D**) As the SS-iOCT operates at longer wavelengths (infrared) distinct from visible surgical light sources, its imaging quality remains unaffected by surgical illumination ((**C**) without endo-illumination; (**D**) with the microscope light source turned off).

Seven eyes (9.7%) with myopic traction maculopathy (MTM) were treated, including three eyes diagnosed with macular schisis, three eyes with macular hole retinal detachment (MHRD), and one eye with foveal detachment (MSRD). Among them, two were male and five were female, with a mean age of 51.9 years (range: 36–73 years) and a mean axial length of 29.4 mm (range: 25.84–32.48 mm). The challenge of performing macular surgery in highly myopic eyes lies in clearly identifying macular structures. Therefore, SS-iOCT proved extremely helpful in MTM surgeries. Moreover, SS-iOCT maintained clear imaging under various conditions, such as in the presence of ICG (indocyanine green) dye, absence of endoillumination, or even when the microscope illumination was turned off (Figure 7).

### 3.7. Application in Subretinal t-PA Injection

Among the 12 cases (16.7%) of submacular hemorrhage (SMH) treated with PPV + ILM peeling + subretinal t-PA (tissue-type plasminogen activator) injection, there were 10 cases of polypoid choroidal vasculopathy (PCV), one case of retinal macroaneurysm, and one case of secondary macular hemorrhage due to laser injury. The cohort included nine males and three females, with a mean age of 59.75 years (range: 19–86 years).

**Figure 8:**
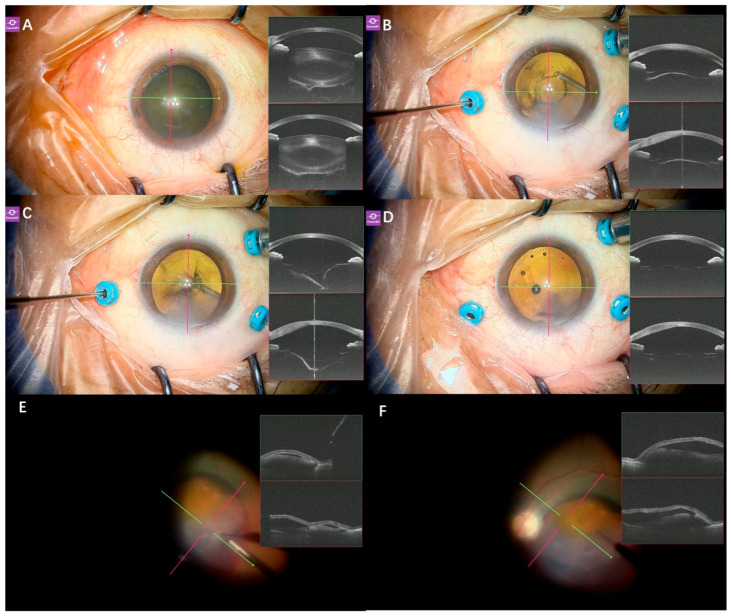
Application of real-time SS-iOCT in a 72-year-old male with PCV and vitreous hemorrhage, who underwent phacovitrectomy. (**A**) SS-iOCT clearly demonstrates the cataract as well as the sand-like opacities within the anterior vitreous. (**B**) SS-iOCT clearly demonstrated the anteriorly displaced posterior capsule adhered to the anterior capsule, as well as the highly reflective anterior hyaloid membrane adherent to the posterior capsule following cataract surgery. (**C**) When the vitrectomy probe engaged the anterior hyaloid membrane, SS-iOCT showed the separation of the anterior hyaloid membrane from the posterior capsule. (**D**) After clearing the anterior hyaloid membrane, viscoelastic agent was injected into the capsular bag. SS-iOCT revealed the absence of the anterior hyaloid membrane, with a distinct separation between the anterior and posterior capsules, appearing as two hyperreflective bands. The posterior capsule remained intact. (**E**) After ILM peeling, SS-iOCT revealed that the 41G needle had touched the retina, causing indentation of the retinal tissue. (**F**) After successful injection, fluctuation of the subretinal blood could be observed, confirming that t-PA had been delivered into the subretinal space.

In one patient with subretinal hemorrhage complicated by vitreous hemorrhage, SS-iOCT clearly demonstrated the anterior hyaloid membrane and sand-like opacities within the anterior vitreous. The turbid anterior vitreous obscured the fundus view; during anterior hyaloid membrane dissection, SS-iOCT assisted in anatomical identification, thereby preventing posterior capsular injury. During subretinal t-PA injection, intraoperative OCT enabled real-time tracking of the 41G needle tip, confirmed that the medication was delivered into the subretinal space, and guided the injection volume (Figure 8).

### 3.8. Application in Intraocular Mass

**Figure 9:**
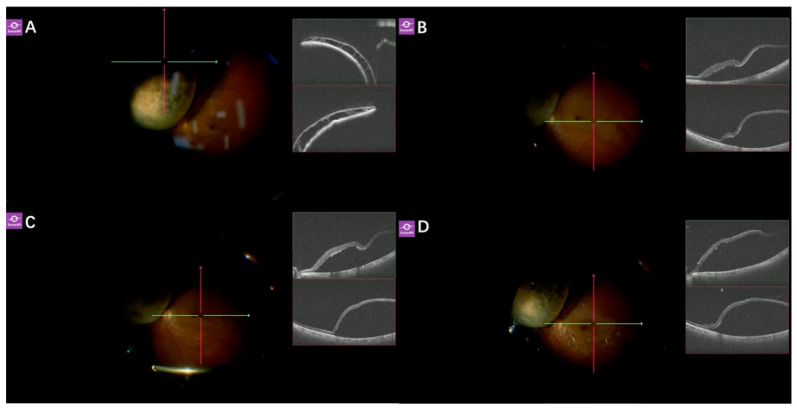
Surgical imaging and real-time SS-iOCT of a 58-year-old male diagnosed with choroidal melanoma undergoing PPV and endoresection. (**A**) Prior to vitrectomy, microscope view reveals an elevated lesion with overlying retinal degeneration and cystoid changes. (**B**) Microscope view shows pigmentary abnormalities in the macular region. SS-iOCT clearly demonstrates subretinal hyper-reflective material, tumor-associated retinal pigmentation (TARP). (**C**) SS-iOCT reveals retinal detachment with TARP and punctate hyper-reflective foci within the subretinal fluid. (**D**) Following TA staining, TA particles are visible on the retinal surface, exhibiting sharper borders and higher reflectivity compared to TARP.

**Figure 10:**
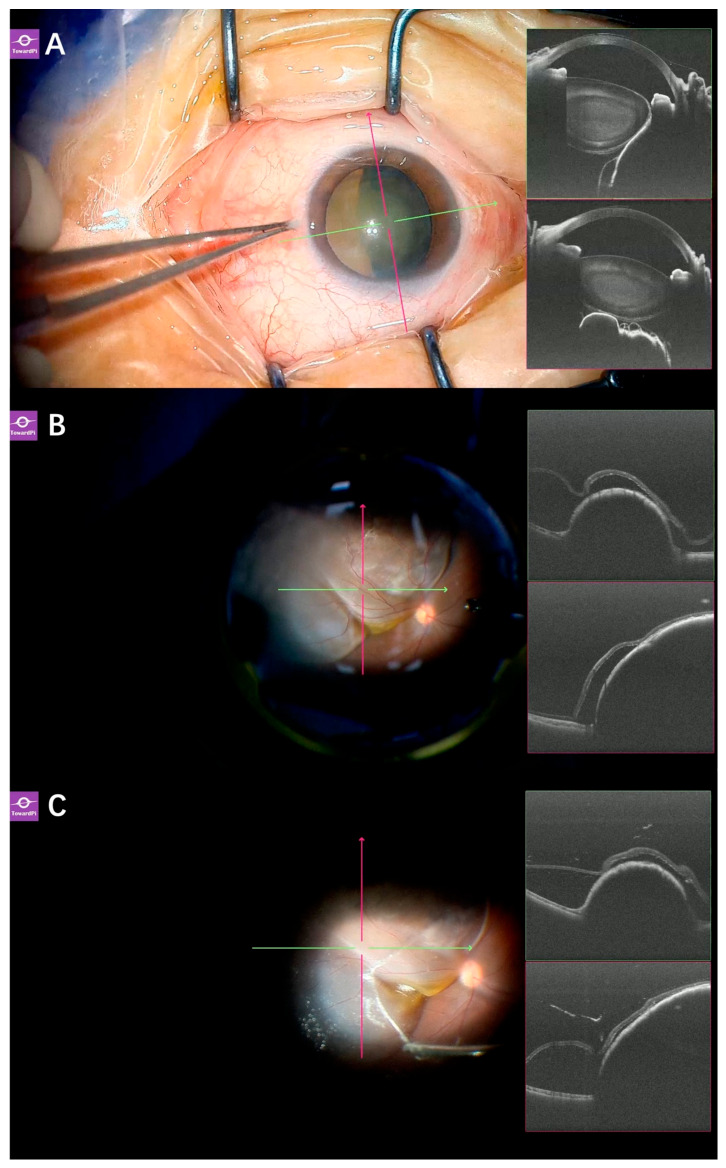
Application of real-time SS-iOCT in PPV and endoresection surgery. (**A**) In a 48-year-old female patient with an intraocular mass, SS-iOCT clearly demonstrated the mass in contact with the lens, as well as the retinal detachment on the surface of the mass. (**B**) In an 18-year-old female patient with uveal melanoma (UM), SS-iOCT revealed retinal detachment on the surface of the tumor. TARP was also observed on SS-iOCT. (**C**) In the same patient, after artificial posterior vitreous detachment (PVD) induction, a Weiss ring was observed, and SS-iOCT clearly visualized the posterior vitreous cortex.

Seven eyes (9.7%) diagnosed with intraocular mass were treated. Among them, five were male and two were female, with a mean age of 54.7 years (range: 18–83 years). The use of intraoperative OCT enabled the real-time visualization and documentation of findings unseen on preoperative exams, capturing valuable data including tumor-associated retinal pigmentation (TARP) and the tumor’s relationship with surrounding tissues (Figure 9 and Figure 10).

## 4. Discussion

In this study, we descriptively report the advantages and feasibility of microscope-integrated 400 kHz SS-iOCT in managing various ophthalmic diseases in a real-world setting. From its role in surgical teaching using routine cases to its application in complex surgical scenarios, we have accumulated experience across a wide spectrum of anterior and posterior segment diseases. Additionally, we provide the first report on the application of SS-iOCT in intraocular tumors.

Intraoperative optical coherence tomography (iOCT) has been increasingly utilized across a wide spectrum of ophthalmic surgeries. In the anterior segment, it provides real-time guidance for cases with corneal opacity [6]. In posterior segment surgery, iOCT is widely applied in various retinal procedures, including retinal detachments [7,8,9], epiretinal membrane (ERM) peeling [10,11], macular hole surgery (e.g., human amniotic membrane grafting [12], VMT [13], optic disc pit maculopathy [14], subretinal gene therapy delivery, pediatric retinal surgeries, high myopia [15] and IOL positioning [16,17]. It provides surgeons with immediate feedback on tissue architecture, instrument–tissue interactions, and the completeness of surgical maneuvers, thereby directly influencing surgical decisions, especially in complex cases [7,15,18].

A historical comparison of intraoperative OCT platforms is summarized in Table 1. Based on our real-world experience with the SS-iOCT system, we have summarized the practical utility and key observations across different surgical indications in Table 2.

We report, for the first time using intraoperative OCT, the detection of subretinal pigmentary alterations secondary to retinal detachment associated with uveal melanoma (UM), which are referred to as tumor-associated retinal pigmentation (TARP). These alterations resemble those previously described in the literature, and electron microscopy has identified them as RPE cells containing melanolipofuscin granules [24,25,26,27].

Regarding pitfalls and difficulties: No significant pitfalls were encountered during the use of intraoperative OCT in this study. The system required an assistant (e.g., another surgeon or trained technician) in the operating room to manually adjust the scan depth and position when needed. However, the operating surgeon could also control the OCT via a foot pedal after initial setup, though this required a brief learning curve. Once the settings were optimized, real-time scanning was achieved without interrupting the surgical workflow.

Regarding effect on surgical duration: The use of intraoperative OCT did not prolong the total surgical time. The scanning process itself was performed in real time and did not require additional pauses beyond the normal surgical sequence. In most cases, image acquisition was integrated seamlessly into the procedure.

Regarding scenarios where swept-source OCT was not feasible: We did not observe any situation in which swept-source OCT imaging was infeasible. Notably, high-quality images were consistently obtained under various intraoperative conditions, including in the presence of air (air-filled vitreous cavity), after indocyanine green (ICG) staining, and even when the microscope light source was turned off.

This study has limitations inherent to its observational design based on a consecutive case series. Moving forward, we aim to accumulate additional cases and clinical experience to further explore svaluable applications of SS-iOCT. Alternatively, future studies may incorporate control groups to quantitatively assess the impact of SS-iOCT on improving surgical success rates. Secondly, this study does not include a direct comparison between preoperative and intraoperative OCT images for each case. One reason is that preoperative OCT images are typically acquired by trained technicians and are primarily intended for diagnostic purposes. In contrast, intraoperative OCT is performed by the surgeon at the time of surgery, allowing targeted imaging of areas that may not have been fully captured or emphasized preoperatively. More importantly, the major advantage of intraoperative OCT lies not in direct comparison with preoperative findings, but in its ability to provide dynamic, real-time feedback during surgical maneuvers and to enable immediate assessment of surgical effects. Thirdly, the OCT image quality is unaffected by potential intraoperative interferences, such as the presence of ICG dye, absence of illumination, or the presence of vitreous or anterior chamber gas bubbles. However, we acknowledge that this observation is qualitative and has not been quantitatively validated. Future prospective studies with blinded, masked grading of image quality across these specific intraoperative conditions are needed to confirm this finding. This report reflects the real-world clinical implementation of an emerging technology, and as its use expands, additional clinically relevant signs and applications are likely to be identified.

## 5. Conclusions

In conclusion, this real-world study demonstrates the broad utility and feasibility of intraoperative SS-iOCT across a diverse spectrum of ophthalmic surgeries. While this observational study highlights the real-world clinical value of an emerging technology, future research will further expand its impact on surgical outcomes and uncover additional applications.

## Figures and Tables

**Table 1:** Technical comparison of microscope-integrated intraoperative OCT platforms.

Parameter	TowardPi BO (This Study)	Zeiss Artevo 800	Leica Proveo 8
Imaging method	SS-OCT (Swept-source)	SD-OCT (Spectral-domain)	SD-OCT (Spectral-domain)
Wavelength	1060 nm	840 nm (typical)/830 nm (minimum)	860 nm
A-scan depth	12 mm	2.9 mm, 5.8 mm	5 mm
Axial resolution	7 μm (at 12 mm depth)	5.5 μm (at 2.9 mm depth)	4 μm (at 2.5 mm depth)
		11 μm (at 5.8 mm depth)	9 μm (at 11 mm depth)
Scan speed	400,000 A-scans/s	27,000 A-scans/s	36,000 A-scans/s
Scan length	20 mm	3–16 mm (1 mm increments)	20 mm
Scan modes	Single-line, cross-line, five-line, radial, 3D, 4D	Single-line, cross-line, five-line, block scan	Single-line, cross-line, five-line, block scan

**Table 2:** Summary of intraoperative OCT’s clinical utility and technical advantages.

Category	Specific Application/Finding	Clinical Benefit/Key Insight
1. Routine procedures & surgical teaching	Anterior segment surgery	Visualizes anterior synechiae and posterior synechiae, and confirms successful goniosynechialysis
	Cataract surgery	Clearly demonstrates corneal incision, hydrodissection, hydrodelineation, and IOL positioning
	Vitrectomy	Enables visualization of posterior vitreous detachment (PVD) and ILM flap covering/plugging
2. Complex cases & intraoperative decision-making	IOL ciliary sulcus fixation	Confirms optimal IOL positioning, helps reduce postoperative astigmatism and improve visual outcomes
	Macular hole surgery	Verifies successful ILM or amniotic membrane insertion; adequate insertion improves closure rates [19,20].
	Pathological myopia	Visualizes macular architecture despite diffuse posterior pole atrophy; effective in axial lengths up to 34 mm
	Choroidal coloboma	Aids in identification of retinal structures where typical choroidal architecture is absent [21].
3. Unique imaging not available preoperatively	Lens–anterior vitreous interface	Captures detailed imaging of an area often overlooked between routine anterior segment and macular OCT exams
	PCV with vitreous hemorrhage	Provides clear visualization during anterior hyaloid removal, helping avoid inadvertent posterior capsule damage
	Intraocular tumors (e.g., uveal melanoma)	Documents retinal changes not typically captured on routine OCT; enables real-time observation of TARP (tumor-associated retinal pigmentation)
4. Technical advantages of SS-iOCT platform	High-speed, high-resolution, deep penetration	Enables transformative intraoperative imaging during vitrectomy for both anterior and posterior segment structures
	Real-time data overlay	Improves surgical ergonomics and efficiency [22,23].
	Mendez Ring display	Enables more precise refractive surgeries (e.g., toric IOL, ICL procedures)

## Data Availability

The raw data supporting the conclusions of this article will be made available by the authors on request.

## Abbreviations

The following abbreviations are used in this manuscript:

OCT	optical coherence tomography
SS-iOCT	swept-source intraoperative optical coherence tomography
MHRD	macular hole retinal detachment
MS	macular schisis
MSRD	macular schisis with foveal detachment
RRD	rhegmatogenous retinal detachment
TRD	tractional retinal detachment
ERM	epiretinal membrane
IOL	intraocular lens
VMT	vitreomacular traction
PCV	polypoid choroidal vasculopathy
MH	macular hole
LMH	lamellar macular hole
SMH	submacular hemorrhage
ILM	internal limiting membrane
t-PA	tissue-type plasminogen activator
TARP	tumor-associated retinal pigmentation
UM	uveal melanoma
PVD	posterior vitreous detachment
ICG	indocyanine green
